# Preventive effect of Curcumin on AD through increasing PS1/E-cadherin/beta-catenin complex mediated by E-cadherin

**DOI:** 10.1186/1750-1326-7-S1-S31

**Published:** 2012-02-07

**Authors:** Xiong Zhang, Wenke Yin, Xiaodong Shi, Yu Li

**Affiliations:** 1 Department of Pathology, Chongqing Medical University, Chongqing, 400016, China; 2 Chongqing Key Laboratory of Neurobiology, Chongqing Medical University, Chongqing, 400016, China; 3 Institute of Neuroscience, Chongqing Medical University, Yuzhong District Yuanjiagang Yixueyuan Road No.1, Chongqing, 400016, China

## Background

The deprivation or abnormality of the molecular function of Wnt/β-catenin triggers the genesis and development of AD, and β-catenin is an important positive mediator in the Wnt/β-catenin signaling pathway. In our previous study, we found that Curcumin could inhibit the expression of β-catenin and prevent AD, but the mechanisms were not fully understood. E-cadherin is a negative mediator in the Wnt/β-catenin signaling pathway, and interacts with β-catenin and PS1 form a trimeric complex, so, we hypothesized that Curcumin prevented AD through increasing PS1/E-cadherin/β-catenin complex by overexpression of E-cadherin.

## Methods

Plasmid APPswe and BACE1-mychis were transiently co-transfected into SHSY5Y cells by Liposfectamin^TM^2000. The cells were treated with Curcumin at 0, 1.25, 5.0, 20.0 μmol/L for 24 h, or with Curcumin at 5.0 μmol/L for 0, and 12, 24 and 48 h for time course assay. Cell lysates were collected for RT-PCR, Western blot assay for detecting the effect of Curcumin on the expression of E-cadherin, β-catenin and PS1. And immunofluorescent staining was carried out for detecting the effect of Curcumin on the expression of PS1/E-cadherin/β-catenin complex. ELISA was carried out to detect the generation of Aβ.

## Results

ELISA results showed that Curcumin reduced markedly the production of Aβ_40/42_. RT-PCR and Western blot results showed that the expression of PS1 and β-catenin at mRNA and protein levels were significantly decreased in the transfected cells treated after treatment; however, the protein expression of E-cadherin was increased (*P* <0.05). Furthermore, all the changes were in a dose and time-dependent manner (*P* <0.05). Immunofluorescent staining results not only confirmed the above changes, but also showed that the PS1/E-cadherin/β-catenin complex was increased.

## Conclusion

Curcumin exerts its preventive effects on AD through increasing PS1/E-cadherin/β-catenin complex by overexpression of E-cadherin.

